# A rare mutation c.1663G > A (p.A555T) in the *MMUT* gene associated with mild clinical and biochemical phenotypes of methylmalonic acidemia in 30 Chinese patients

**DOI:** 10.1186/s13023-020-01632-0

**Published:** 2021-01-07

**Authors:** Lili Liang, Ruixue Shuai, Yue Yu, Wenjuan Qiu, Linghua Shen, Shengnan Wu, Haiyan Wei, Yongxing Chen, Chiju Yang, Peng Xu, Xigui Chen, Hui Zou, Jizhen Feng, Tingting Niu, Haili Hu, Jun Ye, Huiwen Zhang, Deyun Lu, Zhuwen Gong, Xia Zhan, Wenjun Ji, Yongguo Yu, Xuefan Gu, Lianshu Han

**Affiliations:** 1 Department of Pediatric Endocrinology/Genetics, Shanghai Institute for Pediatric Research, Xinhua Hospital, School of Medicine, Shanghai Jiao Tong University, Shanghai, China; 2 Department of Pediatric Endocrinology and Genetics, Children’s Hospital Affiliated to Zhengzhou University, Zhengzhou, China; 3 Center of Neonatal Disease Screening, Jining Maternal and Child Health Care Hospital, Jining, China; 4 Center of Neonatal Disease Screening, Jinan Maternal and Child Health Care Hospital, Jinan, China; 5 Center of Neonatal Disease Screening, Shijiazhuang Maternal and Child Health Care Hospital, Shijiazhuang, China; 6 Center of Neonatal Disease Screening, Shandong Maternal and Child Health Care Hospital, Jinan, China; 7 Center of Neonatal Disease Screening, Hefei Maternal and Child Health Care Hospital, Hefei, China

**Keywords:** Genotype, Methylmalonic acidemia, MMUT gene, Mutation, Newborn screening, Tandem mass spectrometry

## Abstract

**Background:**

Methylmalonic acidemia is an inherited organic acid metabolic disease. It involves multiple physiological systems and has variable manifestations. The primary causative gene *MMUT* carries wide range of mutations, and one of them, c.1663G > A (p.A555T), is considered to be a rare type, which is seen more frequently in Asian than other populations. So far, little is known about the clinical features of patients carrying this mutation. In the present study, we aimed to define the clinical and biochemical features of the patients with this genotype.

**Methods:**

Among 328 *mut* type methylmalonic acidemia patients from multiple hospitals in China, we collected 30 compound heterozygous patients sharing the mutation c.1663G > A (p.A555T) in the *MMUT* gene. Their clinical characteristics and biochemical index were described in detail and compared with methylmalonic acidemia patients without this variant.

**Results:**

Most of these patients were diagnosed via newborn screening (26/30), treated in a timely manner, and kept healthy (24/30). Disease onset occurred in 7 patients. Developmental delay or intellectual impairment occurred in 4 patients. 100% of these patients (29/29) were responsive to Vitamin B12 administration. The blood propionylcarnitine, blood propionylcarnitine/acetylcarnitine ratio, urinary methylmalonic acid, urinary methylcitric acid before and after treatment in c.1663G > A (p.A555T) carrying patients were much lower than those in non-c.1663G > A (p.A555T) carrying patients.

**Conclusion:**

Compared to patients with other mutations in the *MMUT* gene, patients with the c.1663G > A (p.A555T) mutation showed later onset, milder clinical phenotype, lighter biochemical abnormalities, better vitamin B12 responsiveness, lower morbidity, easier metabolic control, and thereby better prognosis. Newborn screening project plays an important role in early diagnosis, treatment, and prognosis of these patients.

## Introduction

Methylmalonic acidemia (MMA) is a series of rare inherited organic acid metabolic disorders. The primary defect occurs in methymalonyl-CoA mutase (MCM) or its cofactor, adenosylcobalamin [1], with the main genetic mode autosomal recessive inheritance. The accumulation of methylmalonic acid and abnormal metabolites causes various clinical symptoms [2]. Implementation of newborn screening in various countries has allowed for the estimation of birth prevalence of MMA and its isolated form. A recent global large size systematic literature review exposed that estimates of MMA (all types) detection rates were 1/126,582, 1/81,967, 1/81,967, and 1/16,556 newborns in Asia–Pacific, Europe, North America and the Middle East and North Africa (MENA) regions, respectively [3]. The incidence of MMA in China varies significantly from region to region and was reported to be 1/38,667 in Shanghai [4], 1/46,531 in Zhejiang province [5], 1/6,032 in Henan province [6], 1/40,166 in Jiangsu Suzhou district [7], 1/16,883 in Jiangsu Xuzhou district [8] and 1/5589 in Shandong Jining district [9]. The expanded screening program for newborns by tandem mass spectrometry (MS/MS) is currently performed in an increasing number of regions of China. The advancing use of MS/MS in newborn screening and identification of clinically suspected cases beneficially serve for proper and timely diagnosis of MMA.

According to the biochemical manifestation, MMA can be classified into two common types: isolated MMA and MMA combined with homocysteinemia. The incidence of isolated MMA was < 1/100,000 newborns in all regions worldwide with the exception of MENA where it approached 6/100,000 newborns [3]. In China, isolated MMA accounts for generally 30% of all types of MMA [5, 8, 9]. The majority of patients with isolated MMA present clinical symptoms and biochemical abnormalities, such as poor feeding, vomiting, poor weight gain, and convulsion within the first few days or months of life. Life-threatening acute metabolic decompensation may occur intermittently, often precipitated by catabolic factors such as infection and stress. The overall prognosis is generally poor, with neurologic and renal impairment [3]. Most cases of isolated MMA are caused by the mutation in the *MMUT* gene, which encodes the protein MCM, and few incidences are due to the changes in other genes, such as *MMAA*, *MMAB*, and others [10]. The *MMUT* gene carries a wide variety of mutations. The mutations spectrum differs significantly in diverse races. For example, c.349G > T (p.E117X), c.385 + 5G > A (IVS2 + 5G > A), c.1106G > A(p.R369H), c.1481T > A (p.L494X), and c.2179C > T (p.R727X) are five relatively frequent mutations in Japan [11]. Indians have many kinds of mutations and c.1863A > T (p.K621N), c.1943G > A (p.G648D), and c.1889G > A(p.G630E) are relatively frequent [12]; c.322C > T (p.R108C) was identified to be frequent in Hispanic patients, while c.2150G > T (p.G717V) was identified as frequent in black patients [13]. In China, the most common mutations include c.729_730insTT(p.D244Lfs*39), c.1106G > A (p.R369H), c.323G > A (p.R108H), and c.1107dupT (p.T370Yfs*22) [8, 14]. The c.1663G > A (p.A555T) mutation in the *MMUT* gene is relatively rare, which is so far reported only in a few cases [7, 15–18]. Limited information is available on the clinical and biochemical characteristics of patients carrying this mutation. In the present study, we examined 30 isolated MMA patients carrying this mutation from multiple hospitals during the last 15 years. We performed a retrospective chart review of their clinical data in detail, including molecular diagnosis, metabolites, treatment, and outcomes, and compared them with those of patients carrying other mutations in the *MMUT* gene, in order to investigate the clinical features and the potential relationship between this genotype and phenotype for the specific mutationc.1663G > A (p.A555T).

## Methods

### Patients

From 2004 to 2019, a total of 1799 MMA patients were diagnosed and treated at multiple hospitals in China. Among them, 328 cases were caused by the *MMUT* gene mutation. We searched for cases carrying the mutation c.1663G > A (p.A555T) with in these patients. As a result, a total of 30 patients (9.15%) were collected, most of which were compound heterozygous with missense, nonsense, frameshift variants or exon deletion in combination with the c.1663G > A (p.A555T).We consider them here after as “c.1663G > A group”. To match the patients carrying c.1663 G > A and “another mutation” in another allele, we selected another 36 MMA patients sharing the some “another mutation” with c.1663 G > A group, as paired control group (non-c.1663G > A group). We compared the clinical and biochemical phenotypes of patients from the two groups. Written informed consent was obtained from the parents of study participants. This study was approved by the Ethics Committee of Xinhua Hospital Affiliated to Shanghai Jiao Tong University School of Medicine (approval ID: XHEC-D-2020-024).

### Detection of metabolites

Blood levels of acylcarnitines, including propionylcarnitine (C3) and acetylcarnitine (C2) were detected by MS/MS (API 4000, American Bio-Systems Inc) using blood filter papers. Urinary organic acids, including methylmalonic acid and methylcitric acid were measured by gas chromatography-mass spectrometry (GC–MS) (Shimadzu Limited, QP2010).

### *MMUT* gene mutation detection and evaluation

*MMUT* gene test was performed by Sanger sequencing or high-throughput next generation sequencing. The mutation was identified by the normal human *MMUT* sequence as a reference (GenBank, NC_000006.12). We used the ClinVar database, the HGMD database and the former literatures to identify whether the mutations had been reported. The pathogenicity of the missense mutation was predicted by the Mutation Taster, PolyPhen-2, Proven and SIFT software. Clustal Omega and HOPE website (https://swissmodel.expasy.org) were used to show the position the mutation occurred in the protein. The HOPE website was also used to establish a crystal structure of human MCM protein with mutation, and then evaluated the potential impact of the mutation on the protein structure.

### Treatment

The treatment of MMA varies with different vitamin B12 responsiveness of the patients. Generally, vitamin B12 responsive patients are treated by vitamin B12, L-carnitine, and low isoleucine, valine, threonine, methionine diet, while vitamin B12 unresponsive patients are treated by L-carnitine and the special diet [19]. Our assessment of the efficacy of vitamin B12 for these patients was based on vitamin B12 loading test and the therapeutic effect of vitamin B12 during the treatment process. Being vitamin B12 responsive is defined as a reduction of more than 50% in the C3/C2 ratio and methylmalonic acid content after vitamin B12 loading test, compared with those before treatment. If the blood C3/C2 ratio and urine methylmalonic acid are decreased but less than down to 50% after vitamin B12 loading test, it is deemed to be “partly responsive” [20]. The judgment standard was also referred to evaluate the therapeutic effect of B12 in patients who did not undergo the loading test.

### Statistical analysis

The normally distributed measurement data were statistically evaluated by using Student t-test, while non-normally distributed data were analyzed by Wilcoxon rank-sum test. The comparison of rates was managed by Chi-square test. Statistical analyses were performed using the GraphPadPrism 5 software (GraphPad Software Inc., San Diego, CA, USA). The p-value of < 0.05 was considered as a significant difference between two groups.

## Results

### Clinical features of the patients

#### Clinical features of c.1663G > A group

The detailed information on each patient carrying c.1663G > A (p.A555T) mutation is summarized in Table 1. Up to now, their median age was 2.8 years old, ranging from seven months to 14 years old. Among these patients, 26 cases were diagnosed by using a positive newborn screening, 2 patients were diagnosed because of onset of the disease (P18, P30), and 2 cases (P9, P13) were diagnosed because of sibling MMA diagnosis (P8, P12). Only seven cases encountered disease onset, among which four cases were subjected to newborn screening (P15, P19, P24, P29) and 3 cases were not (P9, P18, P30).

**Table 1:** Clinical characteristics of the patients carrying c.1663G > A (p.A555T)

Case no	Sex	Age	NS	Age of beginning to treat	Disease onset	Age at onset	Current health status	Hydroxocobalamin effective
P1	F	13 ys	Yes	1 m	No	–	Healthy	Yes
P2	M	5 ys	Yes	4 ms	No	–	Healthy	Yes
P3	M	3 ys	Yes	3 ms	No	–	Healthy	Yes
P4	F	3 ys	Yes	1 m	No	–	Healthy	Yes
P5	M	1 y	Yes	3.7 ms	No	–	Healthy	Yes
P6	M	11 ms	Yes	1 m	No	–	Healthy	Yes
P7	M	1 y	Yes	1 m	No	–	Healthy	Yes
P8	F	2.5 ys	Yes	1 m	No	–	Healthy	Yes
P9	M	4 ys	No	23 ms	Yes	DD	Intellectual impairment	Yes
P10	F	4 ys	Yes	1.7 ms	No	–	Healthy	Yes
P11	F	8 ms	Yes	1 m	No	–	Healthy	Yes
P12	M	7 ms	Yes	1 m	No	–	Healthy	Yes
P13	F	3.4 ys	No	3ys	No	–	Healthy	Not used
P14	F	3 ys	Yes	2 ms	No	–	Healthy	Yes
P15	F	4 ys	Yes	1y	Yes	DD	Intellectual impairment	Yes
P16	M	3 ys	Yes	1.3 ms	No	–	Healthy	Yes
P17	M	2 ys	Yes	1.3 ms	No	–	Healthy	Yes
P18	M	8 ys	No	4.5ys	Yes	DD	Intellectual impairment	Yes
P19	F	3.5 ys	Yes	1 m	Yes	1.8y	Healthy	Yes
P20	M	4.2 ys	Yes	4 ms	No	–	Loss of follow up	Yes
P21	M	3 ys	Yes	1.4 ms	No	–	Healthy	Yes
P22	M	2 ys	Yes	1 m	No	–	Healthy	Yes
P23	F	1 ys	Yes	4 ms	No	–	Healthy	Yes
P24	M	1.6 ys	Yes	1.3 ms	Yes	DD	Developmental delay	Yes
P25	F	2.6 ys	Yes	20 ds	No	–	Healthy	Yes
P26	F	2.5 ys	Yes	2 ms	No	–	Healthy	Yes
P27	F	1 ys	Yes	1 m	No	–	Healthy	Yes
P28	M	2 ys	Yes	1 m	No	–	Healthy	Yes
P29	M	2 ys	Yes	16 ms	Yes	16 ms	Healthy	Yes
P30	M	14 ys	No	2.5 ms	Yes	1 m	Loss of follow up	Yes

Case no	On presentation				After treatment				Mutation 1	Mutation 2
	C3	C3/C2	Methylmalonic acid	Methylcitric acid	C3	C3/C2	Methylmalonic acid	Methylcitric acid		
P1	7.92	0.69	566.23	4.38	3.29	0.26	22.40	1.27	c.1663G > A, p.A555T	c.729_730insTT, p.D244Lfs*39
P2	6.06	0.31	2.31	1.92	3.88	0.23	4.55	1.75	c.1663G > A, p.A555T	c.729_730insTT,p.D244Lfs*39
P3	4.89	0.33	4.62	–	2.967	0.16	<1	0.27	c.1663G > A, p.A555T	c.729_730insTT,p.D244Lfs*39
P4	6.98	0.69	84.50	–	6.781	0.354	47	1.1	c.1663G > A, p.A555T	c.729_730insTT,p.D244Lfs*39
P5	4.95	0.32	18	1.08	2.51	0.08	9.44	<1	c.1663G > A, p.A555T	c.729_730insTT,p.D244Lfs*39
P6	7.88	0.49	27	1.1	8.52	0.16	30	1.62	c.1663G > A, p.A555T	c.729_730insTT,p.D244Lfs*39
P7	3.8	0.52	228.11	9.68	1.83	0.1	2.1	<1	c.1663G > A, p.A555T	c.729_730insTT,p.D244Lfs*39
P8	6.67	0.69	94.31	–	2.20	0.17	0.90	–	c.1663G > A, p.A555T	c.1106G > A,p.R369H
P9	3.86	0.34	6.2	0	3.3	0.25	0.3	<1	c.1663G > A, p.A555T	c.1106G > A,p.R369H
P10	7.11	0.72	124.80	–	2.15	0.28	2.06	0.36	c.1663G > A, p.A555T	c.1106G > A,p.R369H
P11	5.82	0.18	20.09	2.2	1.31	0.07	1.32	0.56	c.1663G > A, p.A555T	c.1106G > A,p.R369H
P12	4.63	0.23	34.6	1.4	1.903	0.113	18.9	–	c.1663G > A, p.A555T	c.2131G > T,p.E711X
P13	3.97	0.27	5.7	0.8	6.3	0.22	15	0.9	c.1663G > A, p.A555T	c.2131G > T,p.E711X
P14	5.02	0.38	–	–	3.8	0.2	–	–	c.1663G > A, p.A555T	c.2131G > T,p.E711X
P15	9.72	0.51	38.96	1.2	7.17	0.26	4.28	0.53	c.1663G > A, p.A555T	c.424A > G,p.T142A
P16	4.86	0.37	9.27	1.33	2.188	0.09	<1	0.11	c.1663G > A, p.A555T	c.424A > G,p.T142A
P17	8.65	0.7	133.91	2.26	5.87	0.2	–	–	c.1663G > A, p.A555T	c.494A > G,p.D165G
P18	3.01	0.19	43.77	–	2.46	0.11	1.1	<1	c.1663G > A, p.A555T	c.613G > A,p.E205K
P19	7.81	0.92	100.08	3.78	5.42	0.3	3	0.13	c.1663G > A, p.A555T	c.626dupC,p.K210X
P20	4.13	0.69	63.82	0.42	4.30	0.30	85.17	4.2	c.1663G > A, p.A555T	c.755_756insA,p.H252Qfs*6
P21	6.76	0.62	286.19	4.22	2.92	0.06	12.77	1.01	c.1663G > A, p.A555T	c.914T > C,p.L305S
P22	6.54	0.33	92.69	2.58	1.96	0.06	13.4	0.6	c.1663G > A, p.A555T	c.1207C > T,p.R403*
P23	5.94	0.45	43.25	–	2.384	0.149	3.25	<1	c.1663G > A, p.A555T	c.1233_1235delCAT,p.I411-
P24	5.85	0.39	22.31	1.5	7.26	0.15	2.93	0.63	c.1663G > A, p.A555T	c.1280G > A,G427D
P25	4.1	0.34	8.5	0	7.41	0.2	33	1.1	c.1663G > A, p.A555T	1679G > A,C560Y
P26	2.4	0.32	45.6	1.07	1.78	0.1	<1	<1	c.1663G > A, p.A555T	c.2009G > T,p.G670V
P27	5.82	0.30	28.80	2.16	4.426	0.133	3.79	<1	c.1663G > A, p.A555T	E13 deletion
P28	4.71	0.41	29.7	0.7	4.93	0.2	77.7	2	c.1663G > A, p.A555T	Not detected
P29	4.67	0.34	174.7	0.89	3.16	0.13	7.25	<1	c.1663G > A, p.A555T	Not detected
P30	6.55	0.85	–	–	1.85	0.31	–	–	c.1663G > A, p.A555T	Not detected

M, male; F, female; NS, Newborn Screening; DD, progressive developmental delay; y, year; m, month; d, day

Typical reference range of C3 in the blood:0.50–4.00 μmol/L; Typical reference range of C3/C2 in the blood:0.04–0.25; Typical reference range of methylmalonic acid in the urine:0–4 mmol/mol creatinine; Typical reference range of methylcitric acid in the urine: 0–0.8 mmol/mol creatinine

–, not available

#### Clinical manifestation

Among the seven cases with a symptomatic presentation, 3 cases showed acute disease onset (P19, P29, P30), while four cases presented developmental delay or intellectual impairment progressively, without acute symptoms (P9, P15, P18, P24). As for the three cases with acute disease onset, the symptoms showed no specificity, including difficult feeding, vomiting, diarrhea, muscle weakness, lethargy, and convulsion. The median age of disease onset was 16 months old, ranging from three days to 22 months old.

The patient P19 was diagnosed by newborn screening and was treated by L-carnitine since one month of age. An acute attack of metabolic acidosis induced by upper respiratory tract infection, showing the symptoms of vomiting, diarrhea, lethargy, and muscle weakness happened at 22 months of age. After symptomatic treatment, the patient gradually recovered in two weeks. P29 had undergone newborn screening at birth. However, the individual did not accept treatment until a disease attack induced by respiratory tract infection at 16 months old, manifested with fever, vomiting, diarrhea, muscle weakness, lethargy, convulsion, and coma. Auxiliary examination indicated metabolic acidosis. P30 was observed to present difficult feeding, poor weight gain, muscle weakness, and metabolic acidosis at 1 month of age.

As for the four patients that showed progressive developmental delay or intellectual impairment, P9 was diagnosed because the younger sibling (P8) was confirmed with MMA in newborn screening, and then, detection of the gene confirmed the diagnosis of P9. A mild developmental delay was observed when diagnosis was confirmed at 23 months old. The patient could not speak until the age of 26 months. The patient P15 showed developmental delay and could not walk until the age of 16 months. P18 was not subjected to newborn screening at birth 8 years ago. The individual could not walk until 24 months old, could not speak until 36 months old, and was diagnosed with MMA at 57 months old because of intellectual impairment. P24 was diagnosed by newborn screening and was treated irregularly. This patient presented progressive developmental delay without attack of metabolic acidosis. Furthermore, the child could not walk properly at the age of 17 months.

#### Treatment and vitamin B12 responsiveness

All the 30 patients accepted treatment after diagnosis. More than half of patients (16/30) received the treatment before being two months old. Most patients were compliant with B12 treatment. Up to December 2019, except for P1 whose parents rejected further treatment after 4 months of treatment, and two patients (P20, P30) who was lost of follow-up, all the other 27 patients received treatment with hydroxocobalamin and/or L-carnitine. A total of nine patients accepted the intramuscular injection with vitamin B12 only, with hydroxocobalamin the single dose of 1–10 mg, and the frequency of once every two days to once every two weeks. A total of 13 patients accepted oral L-carnitine only, with the dose of 50–100 mg/(kg·d). A total of five patients accepted the treatments with both hydroxocobalamin and L-carnitine, with similar doses as above. Nearly half of the patients were maintained on the specialized diet therapy.

The treatment of the seven cases with symptomatic presentation were shown as follows. P9 were treated with L-carnitine and hydroxocobalamin in the first year after  diagnosis and then with hydroxocobalamin only. P15 was diagnosed by newborn screening and was adhered to a low protein diet. The vitamin B12 and L-carnitine were added until 1 year old. P18 has adhered to the treatment with L-carnitine and hydroxocobalamin regularly after  diagnosis. The patients P19 and P24 were diagnosed by newborn screening and treated with L-carnitine and hydroxocobalamin since the age of about one month. L-carnitine was kept at a dose of 100 mg/(kg·d) routinely. The hydroxocobalamin treatment for P24 was stopped unexpectedly when the patient was two months old. As for the patient P29, hydroxocobalamin and L-carnitine were used immediately at the disease onset. Of note, this patient recovered quickly and showed no attacks after treatment. As for P30, Vitamin B12 and L-carnitine were used before the patient gave up the treatment at 2.5 months of age.

With regard to vitamin B12 responsiveness, nine patients have completed the vitamin B12 loading test in our clinic, eight with hydroxocobalamin and one with methylcobalamin (P12). According to C3/C2 ratio and methylmalonic acid before and after the loading test, five cases met the completely responsive type, with C3/C2 ratio and methylmalonic acid decreased more than 50%; three cases met the partly responsive type, with C3/C2 ratio and methylmalonic acid decreased but less than 50%; one case (P16) had inconsistent changes trend in C3/C2 ratio (decrease) and methylmalonic acid (increase).

There were remaining 21 patients did not undergo vitamin B12 loading test. There were two reasons: (1) some patients were transferred to our clinic after vitamin B12 treatment had began in other hospitals. (2) The C3/C2 ratio or methylmalonic acid was as low as normal range or slightly above the normal range. Except for P13 who never used vitamin B12, we evaluated of the therapeutic effect of B12 for the other 20 patients, by referring to the judgment standard of the vitamin B12 loading test. The C3/C2 ratio and methylmalonic acid of the patients on the most recent were compared with the two indexes on presentation. As results, 11 cases showed C3/C2 ratio and methylmalonic acid decreased by more than 50% (11/21); six cases showed C3/C2 ratio and methylmalonic acid decreased but less than 50% (6/21); three case (P6, P25, P28), as well as P13, had inconsistent changes trend in C3/C2 ratio (decrease) and methylmalonic acid (increase).

Because of many confounding factors, such as health status, treatment situation, urine concentration, the diet on the detect day and so on, the urine metabolites fluctuates easily. We consider the changes in blood indicators (C3/C2 ratio) are more meaningful. So we deemed the four (P16, P6, P25, P28) patients with decreased blood C3/C2 ratio and increased urine methylmalonic acid were also responsive to vitamin B12. Therefore, all the nine patients finished the loading test was responsive to vitamin B12tand all the 20 patients received vitamin B12 treatment responded well. There was an obvious overall trend that vitamin B12 treatment is highly effective for these patients (100%, 29/29).

#### Prognosis

As for the current health condition, following up until December 2019, 2 patients could not be followed up (P20, P30). Twenty-four patients (24/30, 80%) were healthy and lived a normal life asymptomatically. Four patients showed progressive developmental delay or intellectual impairment (P9, P15, P18, and P24). P15 and P24 were diagnosed in newborn screening while P9 and P18 did not undergo newborn screening. The Gesell developmental schedule scores of P9 at 22 months old were gross motor 86, fine motor 66, adaptive 93, language 44, personal-social 62. Similarly, the Gesell developmental schedule scores of P15 at 25 months old were gross motor 65, fine motor 63, adaptive 58, language 69, personal-social 56. The Gesell developmental schedules scores of P24 at 17 months old were gross motor 78, fine motor 69, adaptive 78, language 49 and personal-social 49. After treatment, the intelligence of P18 improved slowly. The WISC developmental schedule scores of this patient at 7.2 years were verbal IQ 44, performance IQ < 40, and total IQ < 40.

The patients P19 and P29 who had experienced disease onsets display currently normal intelligence after treatment. The diagnosis of MMA was achieved, and vitamin B12 was used when P30 was 2.5 months old. Unfortunately, individual’s parents gave up the treatment at 3 months old and we failed to confirm the current health status of P30.

### Clinical features of non-c.1663G > A group

The detailed information on the enrolled patients of the control group is summarized in Table 2. There were 23 boys and 13 girls in the control group, with a median age of 2.9 years old, ranging from 12 months to 12.5 years old. Twenty-two patients did not perform MS/MS expanded newborn screening and were diagnosed because of the onset of the disease. Further,14 patients were diagnosed by newborn screening, in which 10 patients showed disease onset during their next treatment. The median age for disease onset of these patients was 3 months old. The symptoms were manifested in varied forms, including difficult feeding, vomiting, diarrhea, poor weight gain, muscle weakness, dyskinesia, lethargy, convulsion, coma, mental retardation, jaundice, anemia, metabolic acidosis, and progressive developmental delay. All the 36 patients accepted treatment after diagnosis. Their median age of beginning treatment was 2 months old. Except for 1case lost during the follow up (C17) and 3 cases of death prior to vitamin B12 treatment (C1, C7, and C8), the remaining 32 patients were subjected to the vitamin B12 loading test. It was seen that 12 patients were responsive to vitamin B12, while the other 20 patients were unresponsive to vitamin B12, yielding a total vitamin B12 responsive rate of 38%, which is significantly lower than that for the c.1663G > A group. As for the current health condition under treatment, 24 patients showed developmental delay or intellectual impairment (67%), five patients are living healthy lives asymptomatically, and six patients died from disease onset at ages ranging from seven days to 18 months.

**Table 2:** Clinical characteristics of the patients in control group

Case no.	Sex	Age	NS	Age of beginning to treat	Disease onset	Age at onset	Current health condition	Hydroxocobalamin effective
C1	M	–	No	5ds	Yes	3ds	Died (7ds)	Unevaluated
C2	M	2 ys	No	7ds	Yes	3ds	Intellectual impairment	No
C3	M	4.1 ys	Yes	1 m	Yes	3ds	Intellectual impairment	No
C4	M	2.9 ys	Yes	1 m	No	–	Healthy	Yes
C5	M	2 ys	No	20ds	Yes	3ds	Intellectual impairment	No
C6	M	2.8 ys	No	15 ms	Yes	3 ms	Intellectual impairment	Yes
C7	M	–	No	3ds	Yes	3ds	Died (10ds)	Unevaluated
C8	M	–	No	2ds	Yes	3ds	Died (7ds)	Unevaluated
C9	F	12 ys	No	20 ms	Yes	20 ms	Intellectual impairment	Yes
C10	F	7 ys	No	12 ms	Yes	12 ms	Intellectual impairment	No
C11	F	–	No	2.5 ms	Yes	3ds	Died (17 ms)	No
C12	F	4 ys	No	16 ms	Yes	16 ms	Intellectual impairment	No
C13	M	2.6 ys	Yes	2 ms	Yes	17 ms	Intellectual impairment	No
C14	F	2 ys	Yes	20ds	Yes	12 ms	Healthy	No
C15	F	1.5 ys	Yes	7ds	Yes	3ds	Developmental delay	Yes
C16	M	1.5 ys	Yes	1.3 ms	Yes	PDD	Developmental delay	No
C17	M	2 ys	Yes	2 ms	No	–	Loss of follow up	Unevaluated
C18	M	7.5 ys	No	8 ms	Yes	6 ms	Intellectual impairment	No
C19	M	5.5 ys	No	7ds	Yes	3ds	Intellectual impairment	Yes
C20	F	1 y	Yes	3 ms	Yes	7ds	Developmental delay	No
C21	M	9.5 ys	No	7 ms	Yes	7ds	Intellectual impairment	No
C22	F	1.9 ys	Yes	1 m	No	–	Healthy	Yes
C23	M	3.3 ys	No	7ds	Yes	3ds	Died (18 ms)	No
C24	F	13 ys	No	46 ms	Yes	46 ms	Healthy	No
C25	M	2.5 ys	Yes	20ds	Yes	6 ms	Intellectual impairment	No
C26	M	–	No	2 ms	Yes	3ds	Died (5 ms)	No
C27	M	4.8 ys	No	17 ms	Yes	12 ms	Intellectual impairment	No
C28	M	2.5 ys	No	6 ms	Yes	3 ms	Intellectual impairment	Yes
C29	M	6 ys	No	12 ms	Yes	12 ms	Intellectual impairment	Yes
C30	F	4.5 ys	No	6 ms	Yes	5 ms	Intellectual impairment	No
C31	F	2.4 ys	Yes	1 m	Yes	15 ms	Intellectual impairment	No
C32	F	3 ys	Yes	2 ms	No	–	Healthy	Yes
C33	M	4 ys	No	4 ms	Yes	4 ms	Intellectual impairment	No
C34	M	1.6 ys	Yes	10ds	Yes	7ds	Developmental delay	Yes
C35	F	1.8 ys	Yes	10ds	Yes	7ds	Developmental delay	Yes
C36	M	3 ys	No	16 ms	Yes	8 m	Intellectual impairment	Yes

Caseno	On Presentation				After treatment				Mutation 1	Mutation 2
	C3	C3/C2	Methylmalonic acid	Methylcitric acid	C3	C3/C2	Methylmalonic acid	Methylcitric acid		
C1	13.33	1.00	301.36	63.64	–	–	–	–	c.729_730insTT,p.D244Lfs*39	c.729_730insTT,p.D244Lfs*39
C2	28.74	1.54	274.80	14.10	41.67	1.28	1937.12	60.23	c.729_730insTT,p.D244Lfs*39	c.729_730insTT,p.D244Lfs*39
C3	23.42	1.02	253.00	17.00	32.81	0.66	195.40	0.87	c.729_730insTT,p.D244Lfs*39	c.467A > T,p.D156V
C4	6.20	0.32	62.00	1.58	7.30	0.37	34.60	2.00	c.729_730insTT,p.D244Lfs*39	c.446A > G,p.D149G
C5	8.68	0.43	258.90	19.80	34.18	1.23	1062.27	30.91	c.729_730insTT,p.D244Lfs*39	c.1399c > T,p.R467*
C6	7.80	0.26	319.00	41.80	21.90	0.47	130.00	4.50	c.729_730insTT,p.D244Lfs*39	c.1286A > G,p.Y429C
C7	19.46	1.30	106.43	8.73	–	–	–	–	c.729_730insTT,p.D244Lfs*39	c.1153_1154delTT, p.L385Afs*6
C8	15.01	1.45	2541.20	84.00	–	–	–	–	c.729_730insTT,p.D244Lfs*39	c.1138G > A,p.G380R
C9	–	–	1426.29	15.33	52.19	0.93	<1	0.30	c.1106G > A,p.R369H	c.1106G > A,p.R369H
C10	15.92	1.03	869.83	10.09	12.75	0.74	701.72	8.56	c.1106G > A,p.R369H	c.1530_1531insTT
C11	10.93	2.10	402.98	40.68	18.96	0.96	<1	33.41	c.1106G > A,p.R369H	c.729_730insTT,p.D244Lfs*39
C12	43.71	0.60	7202.00	–	20.71	0.77	1068.00	5.51	c.1106G > A,p.R369H	c.470T > A,p.V157D
C13	9.46	0.82	448.50	16.20	18.12	0.54	867.31	5.87	c.1106G > A,p.R369H	c.544dupA,p.M182Nfs*29
C14	10.32	0.54	704.12	12.30	47.88	0.89	354.51	3.17	c.1106G > A,p.R369H	c.278G > A,p.R93H
C15	18.00	1.10	523.23	5.52	39.07	1.40	382.44	5.40	c.1106G > A,p.R369H	c.349G > T,p.E117*
C16	21.10	0.79	–	–	29.94	0.95	1929.39	47.28	c.2131G > T,p.E711*	c.1106G > A,p.R369H
C17	2.16	0.37	36.97	3.26	–	–	–	–	c.2131G > T,p.E711*	c.419T > G,p.L140P
C18	4.76	0.36	–	–	26.68	0.53	790.1	3.1	c.2131G > T,p.E711*	c.419T > G,p.L140P
C19	11.81	0.43	9.83	0.00	17.68	0.67	103.00	–	c.424A > G,p.T142A	c.914T > C,p.L305S
C20	7.92	8.81	–	–	63.32	1.06	–	–	c.424A > G,p.T142A	c.544_545insA,p.M182Nfs*29
C21	13.57	0.47	286.85	–	24.77	0.85	162.51	–	c.424A > G,p.T142A	c.419T > C,p.L140P
C22	9.00	1.80	10.90	5.58	24.83	0.44	263.77	<1	c.494A > G,p.D165G	c.1630_1631GG > TA, p.G544X
C23	15.70	0.66	409.00	6.93	14.65	0.62	617.71	22.59	c.494A > G,p.D165G	c.1106G > A,p.R369H
C24	13.19	0.50	–	–	18.12	0.99	256.78	1.17	c.613G > A,p.E205K	c.1677-1G > A
C25	10.87	0.66	14.85	8.39	32.18	0.84	450.46	4.04	c.613G > A,p.E205K	c.982C > T,p.L328F
C26	10.33	0.72	422.84	–	34.03	1.06	495.00	–	c.626dupC,p.K210X	c.1531C > T,p.R511X
C27	21.77	0.53	453.76	10.21	12.15	0.99	145.40	5.54	c.626dupC,p.K210X	c.1084-33delTTTC
C28	18.53	0.55	130.60	12.70	32.09	0.88	0.64	14.80	c.755_756insA,p.H252Qfs*6	c.912-2A > T
C29	4.70	0.38	278.00	1.70	33.07	1.08	1334.22	9.44	c.914T > C,p.L305S	c.970G > A,p.A324T
C30	4.83	0.51	422.73	2.89	16.86	0.91	1254.17	30.46	c.914T > C,p.L305S	c.1677-1G > A
C31	13.50	1.53	431.92	22.45	32.02	1.41	467.17	8.53	c.914T > C,p.L305S	c.2062G > T,p.E688X
C32	15.45	0.22	180.70	–	6.08	0.21	116.03	2.96	c.1233_1235delCAT, p.I410-	c.2080C > T,p.R694W
C33	6.66	0.76	165.30	4.00	12.24	0.81	146.72	0.75	c.1280G > A,p.G42D	c.323G > A,p.R108H
C34	13.61	0.85	284.80	21.20	30.22	1.08	744.63	5.94	c.1280G > A,p.G42D	c.1677-1G > A
C35	9.81	1.08	11,057.34	41.93	27.18	1.42	520.08	5.16	c.1679G > A,p.C560Y	c.1850T > G,p.L617R
C36	26.37	0.59	332.80	0.7	26.62	0.43	335.00	1.80	c.2009G > T,c.2009G > T	c.729_730insTT,p.D244Lfs*39

M, male; F, female; NS, Newborn Screening; PDD, progressive developmental delay; y, year; m, month; d, day

Typical reference range of C3: 0.50–4.00 μmol/L; Typical reference range of C3/C2:0.04–0.25; Typical reference range of methylmalonic acid: 0-4 mmol/mol creatinine; Typical reference range of methylcitric acid: 0–0.8 mmol/mol creatinine

### Comparison of clinical features in two groups

The detailed clinical features comparison of c.1663 G > A group and non-c.1663 G > A group are summarized in Table 3. There were significant differences in presentation and clinical severity between the two groups. The proportions of disease onset were 7/30 (23%) in c.1663 G > A groupand 32/36 (89%) in non-c.1663 G > A group, with a significant difference in the incidence rate between the two groups (*P* < 0.0001). As for the treatment, the vitamin B12 responsive rate was 100% (29/29) in c.1663G > A group and only 38% (12/32) in non-c.1663G > A group (*P* < 0.0001). As for the prognosis, most of the patients carrying c.1663G > A (p.A555T) remained asymptomatic under treatment (24/30). In contrast, most of the patients carrying other mutations manifested developmental delay or intellectual impairment (24/36), and six patients died. A significant difference was also detected in the prognosis of the two groups (*P* < 0.0001). In conclusion, compared with patients carrying other mutations, c.1663G > A (p.A555T)—coding patients exposed lower morbidity, later disease onset, milder clinical phenotype, better vitamin B12responsiveness, and thereby better prognosis.

**Table 3:** Patient cohort characteristics

Variable value	c.1663 G > A group	Non-c.1663 G > A group (n = 36)	Statistical difference
Number of subjects	n = 30	n = 36	
Age (year)	0.58–14.00 (mean 3.44; median 2.80)	1.00–12.5 (mean 3.99; median 2.90; n = 31, the remained 5 cases died)	
Male: female	17/13	23/13	
Newborn screening	26/30 (87%)	14/36 (39%)	
Disease onset	7/30 (23%)	32/36 (89%)	χ2 = 29.09; *P* < 0.0001
Average age of acute onset (month)	0.10–22 (mean 13.00; median 16.00, n = 3)	0.10–46.00 (mean 6.42; median 3.00; n = 30)	
Age of begin treatment (month)	0.70–57 (mean 6.28; median 1.35, n = 30)	0.07–46.00 (mean 5.77; median 2.00; n = 36)	
Vitamin B12 effectiveness	29/29 (100% effective; 1 case has not used Vitamin B12)	12/32 (38% effective; 1 case was lost of followed up and could not be evaluated; 3 cases died before Vitamin B12 use)	χ2 = 26.97; *P* < 0.0001
Current Health Condition	Healthy: 24 cases; developmental delay (< 2y) or intellectual impairment (> 2y): 4 cases; loss of follow up: 2 cases	Developmental delay (< 2y) or intellectual impairment (> 2y): 24 cases; died: 6 cases; healthy:5 cases; loss of follow up: 1 cases	χ2 = 32.79; *P* < 0.0001
C3 level on presentation (μmol/L)	Median 5.82 (2.40–9.72; n = 30)	Median 13.19 (2.16–43.71; n = 35)	U = 121.50; *P* < 0.0001
C3/C2 level on presentation	Median 0.39 (0.18–0.92; n = 30)	Median 0.66 (0.22–8.81; n = 35)	U = 257.50; *P* = 0.0004
Methylmalonic acid level on presentation (mmol/mol creatinine)	Median 41.11 (2.31–566.20; n = 28)	Median 310.20 (9.83–11,057.00; n = 32)	U = 132.00; *P* < 0.0001
Methylcitric acid level on presentation (mmol/mol creatinine)	Median 1.37 (0.00–9.68;n = 22)	Median 11.26 (0–84.00;n = 28)	U = 73.50; *P* < 0.0001
C3 level after treatment (μmol/L)	Median 3.23 (1.31–8.52; n = 30)	Median 26.65 (6.08–63.32; n = 32)	U = 8.00; *P* < 0.0001
C3/C2 level after treatment	0.18 ± 0.02 (0.06–0.35; n = 30)	0.86 ± 0.06 (0.21–1.42; n = 32)	T = 11.56; *P* < 0.0001
Methylmalonic acid level after treatment (mmol/mol creatinine)	Median 4.28 (0.00–85.17; n = 27)	Median 382.40 (0.00–1937.00; n = 31)	U = 77.00; *P* < 0.0001
Methylcitric acid level after treatment (mmol/mol creatinine)	Median 0.53 (0.00–4.20; n = 25)	Median 5.46 (0.00–60.23; n = 28)	U = 65.50; *P* < 0.0001

Typical reference range of C3:0.50–4.00 μmol/L; Typical reference range of C3/C2:0.04–0.25; Typical reference range of methylmalonic acid:0-4 mmol/mol creatinine; Typical reference range of methylcitric acid:0–0.8 mmol/mol creatinine. y, year

### Biochemical features of the patients

As the biochemical makers, the blood C3, blood C3/C2 ratio, urinary methylmalonic acid, urinary methylcitric acid before and after treatment in c.1663G > A and non-c.1663G > A groups are presented in Tables 1 and 2. The comparative results between c.1663G > A and non-c.1663G > A groups are summarized in Table 3.

On the primary state before treatment, C3, C3/C2, methylmalonic acid, methylcitric acid in c.1663G > A group showed a slight increase over the normal range. In contrast, the 4 biochemical indexes of the non-c.1663G > A group showed a prominent increase than the normal range in most patients.

All the 4 biochemical markers in c.1663G > A group before treatment were much lower than those in non-c.1663G > A group, with a significant statistical difference (Tables 1, 2, 3). Similar changes were observed in the two groups after the treatment. The levels of C3, C3/C2 ratio, methylmalonic acid, methylcitric acid in c.1663G > A group after treatment were much significantly lower than those in non-c.1663G > A group. As for c.1663G > A (p.A555T) carrying patients, the levels of C3, C3/C2 ratio, methylmalonic acid, methylcitric acid decreased remarkably after treatment, compared with those before treatment. However, in non-c.1663G > A group, C3, methylcitric acid decreased while C3/C2 ratio, methylmalonic acid increased after treatment, compared with those before treatment, respectively (Tables 1, 2, 3). These data indicated that the therapeutic effect in c.1663G > A (p.A555T) carrying patients was much better than that in non-c.1663G > A (p.A555T) carrying patients.

### Geographical distribution of c.1663G > A (p.A555T)

In order to explore the geographical distribution of the mutation c.1663G > A (p.A555T), we analyzed the origin of the 328 patients harboring mutations in the *MMUT* gene, and calculated the mutation frequency of c.1663G > A (p.A555T) in different regions. As shown in Table 4, the mutation frequency varies notably depending on the region. The population of the Shandong province was found to display the highest mutation frequency, followed by Hebei province and Henan province.

**Table 4:** The mutation frequency of c.1663G > A (p.A555T) in different regions in the conhort

Province	Number of cases carrying c.1663G > A	Number of cases caused by *MMUT* gene mutation	Variation frequency of c.1663G > A
Shandong	14	88	14/176 (7.95%)
Henan	7	56	7/112 (6.25%)
Hebei	3	19	3/38 (7.89%)
Jiangsu	2	24	2/48 (4.17%)
Anhui	1	20	1/40 (2.50%)
Zhejiang	1	19	1/38 (2.63%)
Shanghai	1	13	1/26 (3.85%)
Yunnan	1	1	–
Others districts	0	88	–

### Pathogenic effects of mutation c.1663G > A (p.A555T)

We assessed the potential pathogenicity of the mutationc.1663G > A (p.A555T) by MutationTaster, PolyPhen-2, Proven and SIFT software. It was predicted to be “disease-causing”, “probably damaging”, “deleterious”, and “damaging”, respectively. Furthermore, we evaluated its pathogenicity by the WinterVar database (http://wintervar.wglab.org), according to the ACMG 2015 guideline. It was defined as “likely pathogenic” with the score “PM1 + PM2 + PP3 + PP5”.

The mutationc.1663G > A (p.A555T) leads to analanine into a threonine change at position 555 in the MCM protein. The mutant threonine residue is larger and less hydrophobic than the wild-type alanine residue. The website “HOPE” (https://www3.cmbi.umcn.nl/hope) was used to model the conceivable 3D conformations of wild-type and mutant MCM proteins. The wild-type residue 555 is located in an α-helix ranging from amino acid 548 to 557, and is buried in the core of the protein. The mutated residue threoninedoes not prefer α-helices as a secondary structure for the steric hindrance effect and the loss of hydrophobic interactions, and thereby affects the function of the protein [21].

## Discussion

The *MMUT* gene harbors wide variety of mutations and differs significantly in different races. We have reviewed the allele frequencies from ClinVar and Gnomad. Data from Gnomad showed the following table: The allele frequencies of c.1663G > A in *MMUT* gene is quite rare worldwide, with a total allele frequency of 0.00002786. It has not been discovered in African, Latino, Ashkenazi Jewish, European (Finnish), and South Asian databases while it is relatively common in East Asian (including China), with the allele frequency 0.0003262 (https://gnomad.broadinstitute.org/variant/6-49412365-C-T?dataset=gnomad_r2_1). We have reported a large cohort of 30 MMA patients carrying this mutation, which is consistent with the Genome databases. Apart from our present study, there were another several articles that mentioned this mutation in a few cases, all of which were based on African populations. Wang et al. described three patients, and noted that this was the most common mutation/a hotspot for mutations in *MMUT* in the Chinese population [7]. Chu et al. describes one Hmong patient homozygous for p.A555T and compound heterozygous for CUBN mutations in a study using fibroblasts, suggesting a *mut*^*−*^phenotype and B12 responsiveness [16]. Han et al. described two patients, compound heterozygous condition with missense variants in combination with the c.1663G > A, one of which had mild hyperammonemia at presentation [17]. Kang et al. also mentioned this mutation, however, without detailed description of the clinical information of the carrying patient [18]. Liu et al. presented yet another case of compound heterozygous condition with a different mutation, who had a late symptomatic presentation at age 38 months with acute brain stem encephalitis and myelitis, a devastating complication of *mut* MMA [15]. In the present study, we found the c.1663G > A mutation to be the most frequently occurring in the Shandong, Hebei, and Henan provinces.

Each amino acid has its own specific size, charge, and hydrophobicity. The original wild-type residue and mutant residue often differ in properties. The amino acid site 555 is highly conserved. There were no other residues observed at this position in other homologous sequences. Missense mutations occurred at nearby positions (A535P, C560Y) have been reported in association with MMA. Mutation Taster, PolyPhen-2, Proven, SIFT software and ACMG guideline predicted c.1663G > A (p.A555T) to be pathogenic or probably pathogenic mutation. Besides, the mutation was defined as pathogenic in the Clinvar database based on the reported patient [15]. Furthermore, the 3D-structure model supported the potential impact of the mutation on the crystal structure of the MCM protein. In conclusion, the mutationc.1663G > A (p.A555T) is predicted to impair the function of MCM protein and cause the disease.

Most of the patients in our cohort were diagnosed by newborn screening without symptoms, and a few were clinically confirmed upon onset of the disease. All of them were responsive to the treatment with vitamin B12, and most of them retained asymptomatic thereafter. We suggest that c.1663G > A (p.A555T)-carrying patients caused milder clinical phenotype, better vitamin B12 responsiveness, and better prognosis. There is no specificity in the clinical manifestations of these patients on disease onset, such as vomiting, diarrhea, metabolic acidosis. Despite the milder presentation, complications such as developmental delay or intellectual impairment may also occur, especially if treatment is delayed, such as that observed in case of P9, P15, and P18 in our cohort. Besides, as mentioned above, Liu also has reported a MMA patient carrying c.1663G > A suffered a metabolic stroke of the basal ganglia during a febrile illness [15]. It stressed the point that despite the milder *mut*^−^ phenotype, these patients are important to follow carefully as they remain at risk for severe complications. Therefore, early diagnosis and early treatment is of great importance for these patients, even in individuals with milder, presumably late-onset disease. MS/MS, GC–MS, and gene detection are critical diagnostic methods for the disease and should be promoted in clinically doubtful patients. This is also the group of patients important to pick up at newborn screening before complication occurs. Expanded newborn screening by MS/MS plays critical role in the early diagnosis of the disease. In the non-c.1633G > A group, 22 patients were not diagnosed from newborn screening but from clinical disease onset. Two reasons attributed to this. First, some patients had disease onset several days after birth prior to the newborn screening results, as disease onset age shown in Table 2. Second, some patients performed the routine newborn screening instead of the MS/MS expanded newborn screening, which can pick up MMA but is only available in parts of China. In this point, MS/MS expanded newborn screening should be promoted for a broader range of newborns in China to expose this disease.

As for the biochemical phenotype, patients carrying c.1663G > A (p.A555T) had slightly elevated blood C3, blood C3/C2 ratio, urinary methylmalonic acid, urinary methylcitric acid, which were much lower than those of non-c.1663G > A carrying patients, both before and after treatment. These biochemical markers in c.1663G > A group showed a more pronounced decrease than non-c.1663G > A group during the treatment, also suggesting a better therapeutic effect in c.1663G > A carrying patients. Because of the mild biochemical phenotype, the clinical manifestation is also correspondingly mild. For these patients, gene sequencing is a key assessment in confirming the diagnosis which should be performed in time.

At present, there is no information on the potential underlying mechanism explaining why the mutation c.1663G > A (p.A555T) causes the milder phenotype. One consideration is based on the crystallography of the protein MCM. The *MMUT* mRNA transcript encodes 750 amino acids. Mature human MCM is a homodimer. Each subunit contains an N-terminal extended segment, an N-terminal (βα) 8 TIM barrel domain, a linker region and a C-terminal (βα) 5 Rossmann domain [16]. p.A555T is located in the functionally less important linker region, which does not contribute residues to the catalytic center or the ligand binding pockets, resulting in mut- phenotype or inter allelic complementation. As Chu reported, a 17-month-old Hmong MMA patient carring homozygous c.1663G > A (p.A555T) mutation in the *MMUT* gene was defined to *mut*^−^ type instead of *mut*^0^ type after functional assay in fibroblast [16]. Besides, in a large-scale evaluation of molecular genetic characterization of 151 *mut*^*−*^ type MMA patients by Forny P, deficient alleles in the *mut*^*−*^ subclass were almost exclusively caused by missense mutations, found disproportionately in the C-terminal cofactor binding domain. On the contrary, only half of the *mut*^0^ genotypes were of the missense type. Western blot analysis revealed protein instability as a major mechanism of deficiency in *mut*^*−*^ type MMA [1]. As for the *MMUT* mutations and their relationship to dysfunction and disease, we assume that the milder phenotype induced by c.1663G > A (p.A555T) might also benefit from a missense type of mutation, causing an amino acid substitution rather than deletion of a section of amino acids. Furthermore, the human *MMUT* gene is located on chromosome 6p12.3 and spans over 13 exons. The mutation c.1663G > A (p.A555T) is located in exon 9, which is relatively near to the C-terminal (555/750), which is considered to elicit less effect on the protein functions. It might be another reason for the ameliorated phenotype caused by the mutation.

Three cases (P28, P29, and P30) in our cohort had the classical clinical and biochemical manifestation of MMA. However, we failed to identify their another mutation by DNA sequencing. Two of the three patients had encountered the disease onset at 16 months (P29) and one month old (P30) with severe symptoms, such as muscle weakness, vomiting, lethargy, convulsion and even coma, which suggested that variations existing in their another allele might seriously affect protein function. It is well known that the deletion of exon generally often leads to a more severe phenotype. Therefore, we speculate that exon deletion might exist on another allele in these patients.

## Conclusion

The study present a large cohort of 30 MMA patients carrying a rare pathogenic variant c.1663G > A (p.A555T) in the *MMUT* gene, confers mild biochemical and clinical phenotype and is responsive to B12 treatment. The mild phenotype of the MMA patients with the mutation was indicated by distinct lower morbidity, later disease onset, milder clinical phenotype, lighter biochemical abnormalities, better vitamin B12 responsiveness, easier metabolic control, and better prognosis. Most of our patients carrying c.1663G > A (p.A555T) were diagnosed through the newborn screening, were timely treated, and kept asymptomatic until now. Gene sequencing and expanded newborn screening by MS/MS facilitate early diagnosis and treatment for the disease and should be promoted further.

## Data Availability

All data generated or analysed during this study are included in the published article.

## Abbreviations

GC–MS: Gas chromatography-mass spectrometry
MCM: Methymalonyl-CoA mutase
MENA: Middle East and North Africa
MMA: Methylmalonic acidemia
MS/MS: Tandem mass spectrometry
